# Clinical fundamentals for radiation oncologists

**DOI:** 10.1038/bjc.2011.454

**Published:** 2011-11-22

**Authors:** D M Gujral, C M Nutting

**Affiliations:** 1 Head and Neck Unit, Royal Marsden Hospital, Fulham Road, London SW3 6JJ, UK

Recent developments in new radiotherapy techniques and their applications, along with the use of new agents as radiosensitisers, have resulted in a large volume of data for the busy clinician to assimilate. Treatment options available to cancer patients are ever-increasing and the drive to develop better radiotherapy treatments in terms of technique, delivery, and concomitant systemic therapies, continues. As a consequence, there is a need for practical, up-to-date information that can be easily accessed by radiation oncologists. The author, Hasan Murshed, has presented an upgraded version of Clinical Fundamentals for Radiation Oncology Residents (2006) and retains the style of a revision tool for examination preparation as well as a useful handbook for day-to-day use in the clinic.

The book is conveniently divided into three parts:

(i) The basic science of radiation oncology, with emphasis on radiobiology, physics, and radiation protection; (ii) clinical applications of radiation oncology, presented by tumour site; (iii) palliation in cancer patients. The first section is concise and up-to-date, with good use of illustrative diagrams, and covering all the fundamental basic knowledge required to function as a radiation oncologist. The chapter on radiation protection covers all the important aspects of radiation safety as well as the practical aspects of treatment room design, signage and labelling requirements and equipment, area and personnel monitoring.

Part 2 is conveniently divided into chapters covering the different tumour sites and subsites. Each chapter summarises the management of that particular tumour site, presenting information as bulleted points and tabulating data. There is an annotated bibliography at the end of the chapter covering the major references in that particular field. Each reference is presented with a summary of the main findings of the trial, allowing the reader a quick review of the data. The majority of references cover relevant results from phase III trials from both sides of the Atlantic. Radiotherapy techniques, including IMRT, are summarised with the aid of good quality radiotherapy plans and DVHs. However, the book assumes that the reader has some prior knowledge of patient set-up, immobilisation, and beam arrangement. Most chapters have space allocated at the end of the chapter for the reader to jot down his or her own notes to supplement the information presented. Radiation oncology trainees will find the latest AJCC Cancer Staging Manual, 7th edition of the TNM staging for tumour sites a useful tool for exam preparation. However, the reader should be aware that, while rare tumours such as pineal gland tumours are covered in the text, other tumours such as thyroid cancer and mesothelioma, have been omitted. Thus, for radiation and clinical radiation oncology trainees, another reference textbook is necessary to cover the breadth of knowledge required.

Part 3 is a concise summary of the management of symptoms of cancer and cancer therapy and includes important sections on the management of oncological emergencies such as spinal cord compression, superior vena caval obstruction, and pain due to metastatic bone disease. Chapter 18 covers normal tissue tolerance to therapeutic irradiation, presented in an easy-to-read tabulated format – a useful reference tool for radiation oncologists that could be easily accessed in the busy clinic. This section also covers common medications prescribed for the symptoms caused by cancer and cancer therapy. A summary of the common toxicity criteria at the end of the section is a useful addition.

This book is not designed to provide prescriptive guidelines for management – rather, the evidence is presented in a brief, easy-to-understand format, and it is up to the reader to decide individual patient management. The contributing authors are predominantly based in the United States; consequently, there is a bias towards North American-based practice. However, where differences exist in management between the US and other parts of the world, alternative managements have been suggested to reflect this. For example, in Chapter 11 (Gastrointestinal Cancers), the suggested management of stages Ib–IV gastric cancer is surgery followed by post-operative chemoradiation, a practice adopted mainly in the United States. Peri-operative chemotherapy, widely practiced in the United Kingdom, is mentioned as an alternative. In addition, the author quotes worldwide incidence of certain diseases, then focuses on incidence in the United States.

The author has made some attempt to discuss new radiotherapy techniques such as intensity-modulated radiotherapy. However, in a rapidly evolving field, a short section at the end of Part 2 on emerging technologies, such as image-guided radiotherapy as part of adaptive radiotherapy, stereotactic radiotherapy, and so on, would have been useful and afforded the reader a truly up-to-date synopsis of the radiotherapy techniques on the horizon.

Chemotherapy drugs and doses are summarised, but mechanisms of action and side effects are not presented, so radiation oncology trainees require a further reference textbook to obtain adequate knowledge on systemic therapies. Anatomical diagrams of organs are omitted; therefore, it is assumed the reader already has reasonable knowledge of subsite anatomy.

Overall, the book is a well-written handbook covering the important aspects of radiotherapy. Readers should be aware of the bias towards US-based practice. A further reference textbook is required for the reader seeking a broader understanding of cancer therapy, which includes systemic therapy and emerging radiotherapy techniques.

